# Effect of the Pasta Making Process on Slowly Digestible Starch Content

**DOI:** 10.3390/foods12102064

**Published:** 2023-05-20

**Authors:** Rossella Dodi, Giuseppe Di Pede, Cecilia Scarpa, Valeria Deon, Margherita Dall’Asta, Francesca Scazzina

**Affiliations:** 1 Department of Food and Drug, University of Parma, Parco Area delle Scienze 27/A, 43124 Parma, Italy; 2 Global Nutrition & Wellbeing Unit, Group Research Development & Quality, Barilla G. e R. Fratelli, Via Mantova, 166, 43122 Parma, Italy; 3 Department of Animal Science, Food and Nutrition, Università Cattolica del Sacro Cuore, via Parmense 84, 29122 Piacenza, Italy

**Keywords:** pasta, cereal, starch digestibility, pasta shape, glycemic response, slowly digestible starch

## Abstract

The rate at which starch is digested in the human intestine elicits different glycemic responses and reflects the glycemic index (GI) of foods. In vitro measurement of starch digestibility can reflect the GI of food. Differences in starch digestibility among four durum wheat pasta samples, couscous, and bread were evaluated to better describe the role of the pasta making process in affecting starch digestibility. Statistical differences in RDS (rapidly digestible starch), SDS (slowly digestible starch), and RS (resistant starch) of products were found (*p* < 0.05). As expected, pasta samples showed the highest value of SDS/av starch compared to couscous and bread. Fusilli and cavatelli samples presented the highest SDS/av starch ratio (55.80 ± 3.06% and 53.91 ± 3.50%, respectively), then came spaghetti 49.39 ± 2.83% and penne 45.93 ± 1.19%, while couscous presented the lowest value of SDS/av starch (2.64 ± 0.50%), followed by bread (11.78 ± 2.63%). Our study confirmed that the pasta making process efficiently mediates an increase in SDS/Av starch content, which has been specifically quantified above 40%, therefore strongly related to a lowered glycemic response in vivo. Our results strengthened the concept that pasta is a good source of SDS, which makes it useful for glycemic control.

## 1. Introduction

The rate at which starch present in foods is digested and absorbed within the gastrointestinal tract elicits different glycemic responses, which are usually quantified to calculate the Glycemic Index of foods (GI) [1]. In fact, GI was originally introduced by Jenkins and colleagues to rank carbohydrate-containing foods according to their ability to increase the postprandial glycemic response in comparison to a reference meal [2]. A nutritional classification of foods based on the rate and the extent of starch digestion during in vitro simulated GI digestion could be useful to predict the glycemic response in vivo, and in vitro measurement of starch digestibility can be a good parameter to predict the GI of starchy foods [3]. In fact, according to Englyst’s classification [4], starch can be divided into three classes: RS (resistant starch), RDS (rapidly digestible starch), and SDS (slowly digestible starch). RS represents the fraction, which is not modified and absorbed during the digestion occurring in the small intestine and which is reaching the large intestine where it can be fermented by the intestinal microbiota, producing gases, such as hydrogen, carbon dioxide, methane, and other derived metabolites (short-chain fatty acids). RDS and SDS represent, respectively, the available starch fractions that are rapidly (after 20 min of digestion) and slowly (after 120 min of digestion) released during the simulated in vitro digestion [4]. The rate of starch digestion may be affected by intrinsic food factors, such as the nutritional composition, botanical (e.g., wheat cultivar) and technological aspects (size and shape), production process, and extrinsic food-related factors, such as cooking method applied to foods [5,6,7] and degree of mastication [8]. Moreover, recently, the literature has confirmed that genetics, physical, enzymatic, and chemical modifications can enhance the content of SDS in starchy products [9].

Among cereals, pasta represents an important staple food, contributing to the intake of complex carbohydrates. Compared to other carbohydrate-rich foods, pasta stimulates a lower post-prandial glycemic response, representing an important low GI element of the Mediterranean diet [10,11]. This quality factor depends on the process applied to the wheat semolina obtained after the milling process. During the pasta making process, the extrusion and the subsequent drying step ensure the formation of the highly compact protein network, leading to a more intense starch encapsulation that seems to be involved in reducing the fraction of RDS of native starch in semolina and, consequently, the GI of this type of food [12]. Moreover, the protein structure strengthens during cooking, and it is preserved even after prolonged cooking times [12]. Consequently, this gluten matrix constitutes a barrier for ɑ-amylase within GI tract, thus slowing down starch hydrolysis and subsequent glycemic response [12].

Because of this, different starchy products, made starting from the same raw materials but different processes, may have different GI values. This is the case for two other starchy wheat-based foods commonly consumed within the Mediterranean area, such as couscous and bread [13,14]. In fact, bread is produced by mixing water, milled soft wheat flour, salt and yeast, and, during the making process, the dough mass started to expand through the generation of carbon dioxide gas. The characteristics of the bread structure make the starch present more bioaccessible to digestive enzymes [15] and thus more bioavailable. Similarly, couscous is generally produced with flour, fine cracked wheat (bulgur), semolina, and salt [16]. It is produced through four operations: wet agglomeration, rolling-sieving, steam cooking, and drying [17]. Steam cooking is the critical step in making couscous because it directly contributes to the digestibility of the final products [18]. In fact, during this process, several physical and chemical changes occurred, such as the gelatinization of the starch and the insolubility of gluten proteins [19], which are not structured as a continuous network inside the couscous grain (in contrast with the structure of the durum wheat pasta) [17,18]. Despite the amount of evidence supporting the low GI value of wheat-based pasta, scarce are the studies specifically conducted for investigating the starch bioaccessibility of pasta products compared to other starchy foods commonly consumed as alternatives to pasta (e.g., bread and couscous), all produced starting from the same raw material.

Therefore, the aim of this study was to investigate the effect of pasta production on the SDS content in comparison with other semolina-based products, such as couscous and bread; moreover, different shapes (both short and long) of wheat-based pasta (penne, fusilli, spaghetti, and cavatelli) were analyzed for better understanding the role of different pasta process in affecting starch digestibility.

## 2. Materials and Methods

### 2.1. Samples

In the present study the starch digestibility of 4 durum wheat pasta samples, one sample of bread and one sample of couscous were analysed; in order to avoid any bias deriving from using a different type of ingredients, all the samples were prepared with the same type of semolina. Couscous and semolina for bread preparation were industrially manufactured by Barilla G. e R. F.lli S.p.A and commercially available in Europe. Couscous was prepared by pouring boiling water on couscous (1:1 *v*/*v*), mixing gently, and letting it stand for 5 min before the analysis. Bread was baked using the ingredients and following the procedure described in Table 1 and using a bread machine (“La Panetteria 151936”, Princess Silver).

Four different pasta samples (penne, spaghetti, fusilli, cavatelli—Barilla G. e R. F.lli S.p.A) were analysed in order to evaluate the influence of pasta production and pasta shape on starch digestibility. Penne, fusilli, and cavatelli were short pastas, while spaghetti was a long pasta. To reduce the variation due to different grain harvests and to limit industrial and technological variabilities, the pasta samples were obtained the same year of production from a single factory. After industrial production, all pasta samples were stored as per package instruction. Therefore, once received, the samples were kept at room temperature in a cool and dry place. Each pasta sample was cooked in unsalted boiling water (70 g of pasta in 1 L of boiling water), according to the package instruction. The preparation instructions for all test foods are shown in Table 1.

### 2.2. Determination of RDS, SDS, and RS Fractions

The percentage of SDS, RDS, and RS of tested foods were measured according to the method proposed by Englyst and colleagues [20]. Briefly, 2 g of each product, prepared as described in Table 1 and minced using a mincer with 7 mm holes (Adler Ad 4808, Adler Europe Group, Warsaw, Europe), were weighted into plastic tubes. After the addition of 10 mL of pepsin–guar solution (5 g/L pepsin (P7000, Sigma-Aldrich, St. Louis, MO, USA) and 5 g/L guar (G4129, Sigma-Aldrich, St. Louis, MO, USA) in 0.05 M HCl), the tubes were vortex-mixed and incubated into a shaking water bath (SW23, Julabo®, 77960 Seelbach, German) at 37 °C 180 rpm for 30 min. Five glass marbles and 10 mL of preheated (37 °C) 0.25 M sodium acetate were added, and the tubes were mixed and placed in a water bath for 3 min to equilibrate temperature. An amount of 5 mL of the enzyme mixture was added to each sample, and the samples were incubated in a water bath at 37 °C and 200 rpm. The enzyme mixture was prepared by dissolving into 4 different batches 3.3 g of pancreatin (P7545, Sigma-Aldrich, St. Louis, MO, USA) in 22 mL of distilled water. After centrifugation (3200 rpm for 10 min), 15 mL of supernatant from each batch was collected; 3.6 mL of amyloglucosidase (A7095, Sigma-Aldrich, St. Louis, MO, USA) and 37.5 mg of invertase (I4504, Sigma-Aldrich, St. Louis, MO, USA) were diluted into 3.06 mL of distilled water and added to the supernatant. After 20 min and 120 min, 1 mL of hydrolisate was collected and immediately put on ice. Samples were centrifuged (14,000 rpm for 5 min), and the supernatant was diluted in distilled water (dilution 1:10), and then it was used to determine the total glucose concentration (TG). Free sugar glucose (FSG) was quantified as previously described by Dodi et al. [6].

Glucose released after 20 min of incubation (G_20_), and 120 min of incubation (G_120_),was analysed using an automatic glucose analyser (model 2900, Yellow Springs Instrument Company, Yellow Springs, Ohio, USA). Therefore, rapidly available glucose (RAG), slowly available glucose (SAG), RDS, and SDS were calculated as follows:

RAG = G_20_

SAG = G_120_ – RAG

Available carbohydrate = RAG + SAG

RDS = 0.9 ∗ (RAG – FSG)

SDS = 0.9 ∗ (G_120_ – RAG)

Available starch (Av. Starch) = RDS + SDS

RS = 0.9 ∗ (TG – G_120_)

In vitro digestions were performed in quadruplicate for each sample.

### 2.3. Statistical Analysis

Data are expressed as mean ± SD, unless otherwise stated. Comparisons between the test foods in measures assessed by in vitro methods were performed using one-way analysis of variance (one-way ANOVA) with Tukey HSD as a post hoc test. Statistical analysis was performed using SPSS software (vers. 26, IBM SPSS Statistics, Armonk, NY, USA).

## 3. Results

The values of RDS, SDS, and RS (g/100 g product “as eaten”) for each product are reported in Table 2. After comparing the samples, differences for RDS, SDS, and RS values were observed (*p* < 0.05). As expected, bread showed the highest value of RDS (33.20 ± 0.79 g/100 g), followed by couscous (30.40 ± 0.58 g/100 g). Pasta samples showed a significantly lower values from bread and couscous, with cavatelli having 11.54 ± 0.81 g/100 g of RDS, fusilli having 12.01 ± 1.56 g/100 g of RDS, followed by spaghetti having 14.64 ± 0.69 g/100 g of RDS, and penne having 15.50 ± 0.97 g/100 g of RDS (*p* < 0.05). Considering the SDS content, pasta samples did not show any statistical difference among the different shapes analysed (*p* > 0.05), with SDS ranging from 13.52 ± 1.29 g/100 g for cavatelli, to 15.07 ± 0.16 g/100 g for fusilli, while bread and couscous presented a significantly lower content of SDS than the pasta samples (4.44 ± 1.03 g/100 g, and 0.82 ± 0.15 g/100 g respectively, *p* < 0.05). As expected, RS represents only a minor fraction in these products. After comparing results, bread showed the highest content of RS (1.06 ± 0.05 g/100 g), followed by spaghetti and fusilli, cavatelli, and penne, which presented similar RS content, and couscous had the lowest value.

**Figure 1:**
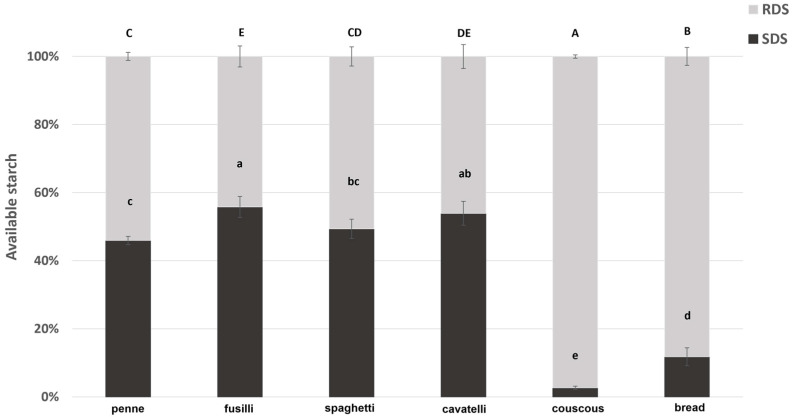
RDS and SDS as % of available starch (mean ± SD) for penne, fusilli, spaghetti, cavatelli, couscous, and bread. Different small letters above the histograms mean statistical differences in SDS/av starch among samples (*p* < 0.05). Different capital letters above the histograms mean statistical differences in RDS/av starch among samples (*p* < 0.05) after one way ANOVA with Tukey HSD post hoc correction.

The % contribution of RDS and SDS as available starch (considered as 100%) is graphically reported in Figure 1. Taking into consideration the ratios of RDS/av starch and SDS/av starch of samples, some statistical differences were observed (*p* < 0.05). The pasta samples showed a higher value of SDS/av starch compared to couscous and bread. Fusilli and cavatelli samples showed the highest ratios of SDS/av starch (55.80 ± 3.06% and 53.91 ± 3.50% for fusilli and cavatelli samples, respectively) and the lowest ratios of RDS/av starch (44.2 ± 3.06% and 46.08 ± 3.50% for fusilli and cavatelli samples, respectively). Spaghetti showed intermediate ratios of both SDS/av starch (49.39 ± 2.83%) and RDS/av starch (50.61 ± 2.83%), followed by penne sample that had the lowest ratio of SDS/av starch (45.93 ± 1.19%) and the highest ratio of RDS/av starch (54.06 ± 1.19%), among pasta samples. Couscous had the lowest value of SDS/av starch (2.64 ± 0.50%), followed by bread (11.78 ± 2.63%). Considering the RDS/av starch content, bread and couscous showed the highest value (88.22 ± 2.63% and 97.46 ± 0.50% for bread and couscous, respectively).

## 4. Discussion

In the present study, the effect of pasta making process on the quality of starch (express as SDS/av starch) has been analysed. Pasta resulted in a mean 39% larger increase in SDS/av starch than bread, and this was 49% higher than couscous. Results obtained confirm that the technological process behind the production of pasta may be responsible for the greater level of SDS, resulting in a significant reduction in rate of starch digestion compared to bread and couscous. Considering that all the analysed pastas, independently from the shape, showed values of SDS/av starch above 40%, they resulted all potentially eligible for bearing the approved health claim on the lowered post-prandial glycemic response in vivo.

In 2011, European Food Safety Authority (EFSA) confirmed that the rate of starch digestibility assessed in vitro in cereal products can be considered a good parameter to predict the in vivo effect on post-prandial glycemic responses. In particular, a cause–effect relationship has been established between the consumption of cereal products that have at least 40% of available starch as SDS and reduced post-prandial glycemic responses [21]. More recent up-to-date evidence also investigated and confirmed the relationship between SDS inclusion within the diet and the decrease in post-prandial glycemic response [9]. Diet characterized by foods exerting a low glycemic excursion may provide a protective effect on chronic diseases [22]. Therefore, due to the low GI and high % of SDS, pasta represents a good candidate to follow a low GI diet [10,23]. During the digestion process of food rich in SDS, glucose is slowly absorbed by passing through the small intestine arriving at the portal vein for the assimilation. Incretin hormones decrease the gastric emptying rate related to satiety and food intake, which can be associated with glycaemic control and weight loss. Therefore, a slow glucose release induced by SDS fraction is accompanied by a low insulin level, which might provide wide health benefits to reduce the risk of diabetes or metabolic syndrome [24]. In fact, one of the major public health challenges is the prevention and management of obesity and diabetes, diseases that are increasing worldwide. The etiology of these pathologic conditions is multi-factorial. However, inadequate dietary habits is recognized as factor contributing to their onset. The recent literature highlighted that slowing down the rate of digestion of glucose from ingested carbohydrate sources helps to contain glycemia, reduces insulin needs, and causes satiety. Low-GI diets elicit a more stable glycemic profile, reducing postprandial hyperglycemia and hyperinsulinemia, as well as attenuating late postprandial rebound in circulating, non-esterified fatty acids (NEFA), all of which are factors that exacerbate these metabolic syndromes. Lower glycemic and insulinemic responses are associated with improved risk profiles, including insulin sensitivity, as well as β-cell function, high-density lipoprotein cholesterol, oxidative status, prothrombotic factors, and endothelial function [25]. SDS-rich foods elicit a moderate postprandial glycemic and insulinemic response, resulting in reducing common chronic diseases related to dietary pattern, such as diabetes, cardiovascular diseases, and obesity. SDS-rich foods may exert these effects by reducing the stress on regulatory systems related to glucose homeostasis. Glucose and insulin concentration rose faster in healthy and type 2 diabetic patients when RDS was digested than when SDS was digested. SDS consumption leads to a low and sustained glycemic and insulinemic response, as well as low NEFA, which can decrease cholesterol. Such a response can contribute to the prevention and treatment of diabetes and the complications of this metabolic syndrome [26].

Despite the positive characteristics described for pasta, a reduction in consumption of this food during the years has been reported. One of the main reasons behind this dietary habit is probably due to the overflowing fake news about carbohydrate-rich foods consumption and gain weight, even though several scientific contributions clearly debunk this myth [27]. Among cereal products, pasta is one of the main sources of SDS, and several studies have shown that pasta products usually have a GI that falls between the low (≤55) and the medium (56–69) range [10,11]. As already mentioned, this can be ascribable to its structure that is the result of successive structural changes occurring in its main components, starch and proteins, throughout the production process. The pasta making process involves three steps: mixing, forming (by extrusion), and drying; finally, cooking in excess water gives pasta its ultimate structure, which is generally described as a compact matrix made of gelatinized starch strongly entrapped in a protein (gluten) network. The encapsulation of starch by the gluten network and the surface area accessible to ɑ-amylase during digestion represent the principal characteristics that have to be taken into account to explain the lowered enzymatic susceptibility of starch in cooked pasta [23]. Moreover, Berti found that gluten free pasta and conventional pasta had in vitro similar starch digestibility, highlighting the importance of technological process instead of the presence of gluten [28]. The technological process employed during pasta making can preserve both nutritional and structural properties. This was clearly evidenced by comparing the SDS/av starch of control foods produced with the same starting semolina (couscous and bread) to the pasta analysed. Couscous showed the lowest ratio of SDS/av starch, and the making process of couscous does not involve kneading and extrusion. However, this is the case for pasta making, but its production involves hydration and steaming before drying, generating a high degree of gelatinized starch and a higher capacity of water absorption during the cooking process [29]. The cooking treatment is able to change the physical and chemical characteristics of the couscous. Moreover, the gelatinization makes starch more bioaccessible to digestive enzymes [29]. Similarly, the different rate of starch digestion in bread is due to the baking process that led to the formation of a porous matrix that is easily destructed, reflecting a higher digestion of starch in vitro [30], despite the durum wheat employed in the baking process. However, since the technological process employed to formulate the different types of pasta used in our study is the same, the difference found in our samples has to be ascribable to different shape of pasta samples. Among pasta samples, penne and spaghetti showed the highest ratio of RDS/av starch, which is ascribable to their structure, showing a higher surface area accessible to α-amylase, while cavatelli and fusilli have a more compact and solid shape, resulting less accessible to enzyme. Our results are in line with Grandfeldt and colleagues, who showed that the pasta size (vermicelli vs. spaghetti) and shape (macaroni vs. spaghetti) seem to be of great importance in starch digestibility. Indeed, the higher surface to weight ratio of vermicelli compared to spaghetti may explain the higher accessibility of α-amylase to starch [31]. Similarly, Wolever et al. found that macaroni produced a significantly higher in vivo glucose response than spaghetti in diabetic subjects [32].

The present study presents some strengths and limitations worth highlighting. The first strength is linked to the samples analysed, all specifically produced starting from the same semolina to avoid any bias deriving from different wheat cultivars, batches, and milling processes used. Moreover, bread was produced without the addition of other ingredients interfering with the SDS and GI of products, such as fats (e.g., oils or lard commonly used for bread production), in order to study only the effect on SDS induced by the different technological process applied. Limitations are certainly linked to the absence of a complete characterization of other technological and molecular parameters that could better describe this in vitro behaviour. Lastly, as already well described in a previous work published [23], in vivo mastication is a key step for mediating a different in vitro and in vivo glycemic response. This work, even if it was based on the EFSA-recognized method for characterizing starchy foods, which used a mincer for mimicking mastication, does not specifically consider inter-individual difference, which may arise depending on the different events occurring in the oral cavity and subsequent starch digestibility.

## 5. Conclusions

The present work supports the evidence that pasta making process is an efficient method to enhance the content of SDS on available starch. Moreover, pasta showed a good content of SDS and a relevant SDS/Av starch ratio, which has been specifically quantified for these samples above 40%, and, therefore, it potentially mediates a reduction in post-prandial glycemia, as described in the scientific opinion on SDS published by EFSA. On the contrary, other starchy foods commonly consumed as alternative to pasta, such as bread and couscous, made with the same durum wheat semolina, presented a scarce content of SDS/av starch, therefore reflecting their higher glycemic response in vivo. Our results strengthened the concept that pasta, despite the size and shape, is a good source of SDS, which makes it useful for the glycemic control associated with several health benefits, including reduced insulin demand, improved blood glucose control, and reduced blood lipid levels. Additionally, the scientific literature has highlighted that the consumption of pasta is involved in decreasing body weight, resulting in optimal carbohydrate-based foods, which is also the case for obese or overweigh individuals. These are important elements for scientifically supporting the consumption of pasta products, considering the recommended portion size and consumption frequency, as they may play an important role in the promotion of health and prevention and management of several chronic diseases.

## Figures and Tables

**Table 1:** Preparation instructions for the test foods.

Test Foods	Ingredients for Food Preparation	Preparation Instructions
Cooked short durum wheat dried pasta_*penne*	- 70 g penne - 1 L water	An amount of 70 g of dried penne pasta was cooked in 1 L of boiling water for 11 min (as per package instructions). Pasta was drained and processed.
Cooked short durum wheat dried pasta_*fusilli*	- 70 g fusilli - 1 L water	An amount of 70 g of dried fusilli pasta was cooked in 1 L of boiling water for 11 min (as per package instructions). Pasta was drained and processed.
Cooked long durum wheat dried pasta_*spaghetti*	- 70 g spaghetti - 1 L water	An amount of 70 g of dried spaghetti pasta was cooked in 1 L of boiling water for 9 min (as per package instructions). Pasta was drained and processed.
Cooked short durum wheat dried pasta_*cavatelli*	- 70 g cavatelli - 1 L water	An amount of 70 g of dried cavatelli pasta was cooked in 1 L of boiling water for 9 min (as per package instructions). Pasta was drained and processed.
Durum wheat semolina home-made *bread* (fresh)	- 500 g durum wheat semolina flour - 10 g sugar - 10 g salt - Dried yeat (1 packet, 7 g) - 300 mL water	All ingredients were added in the bread machine (“La Panettiera”, Princess, Italy), and the “basic (3 h)”, “crust light” settings were selected (program 1). The machine was started the afternoon before the test day. When the bread was ready, it was removed from the machine and left to cool to room temperature (~8 h). When completely cooled, the sides of the loaf were discarded to ensure equal amounts of crust and inner bread. The bread was then processed.
*Couscous*	- 70 g couscous - 80 mL water	Couscous was put in 80 mL of boiling water, stirred with a fork, covered with a lid, and cooked for 5 min. Couscous was stirred again and then processed.

**Table 2:** RDS, SDS, and RS values of products “as eaten”. Data are expressed as mean ± SD.

Samples	RDS (g/100 g)	SDS (g/100 g)	RS (g/100 g)
**Penne**	15.50 ± 0.97 ^c^	13.17 ± 0.72 ^a^	0.56 ± 0.05 ^c^
**Fusilli**	12.01 ± 1.56 ^d^	15.07 ± 0.16 ^a^	0.50 ± 0.02 ^c^
**Spaghetti**	14.64 ± 0.69 ^c^	14.30 ± 0.96 ^a^	0.68 ± 0.04 ^b^
**Cavatelli**	11.54 ± 0.81 ^d^	13.52 ± 1.29 ^a^	0.55 ± 0.07 ^c^
**Couscous**	30.40 ± 0.58 ^b^	0.82 ± 0.15 ^c^	0.40 ± 0.05 ^d^
**Bread**	33.20 ± 0.79 ^a^	4.44 ± 1.03 ^b^	1.06 ± 0.05 ^a^

RDS: rapidly digestible starch; SDS: slowly digestible starch; RS: resistant starch; Data in the same column with different superscript indicate significant difference at *p* < 0.05, after one way ANOVA with Tukey HSD post hoc correction.

## Data Availability

Data is contained within the article.
